# *In Vivo* Antioxidant and Hypolipidemic Effects of Fermented Mung Bean on Hypercholesterolemic Mice

**DOI:** 10.1155/2015/508029

**Published:** 2015-05-17

**Authors:** Swee Keong Yeap, Boon Kee Beh, Wan Yong Ho, Hamidah Mohd Yusof, Nurul Elyani Mohamad, Norlaily Mohd Ali, Indu Bala Jaganath, Noorjahan Banu Alitheen, Soo Peng Koh, Kamariah Long

**Affiliations:** ^1^Institute of Bioscience, Universiti Putra Malaysia, 43400 Serdang, Selangor, Malaysia; ^2^Department of Bioprocess Technology, Faculty of Biotechnology and Biomolecular Sciences, Universiti Putra Malaysia, 43400 Serdang, Selangor, Malaysia; ^3^School of Biomedical Sciences, The University of Nottingham, Malaysia Campus, Jalan Broga, 43500 Semenyih, Selangor, Malaysia; ^4^Department of Cell and Molecular Biology, Faculty of Biotechnology and Biomolecular Sciences, Universiti Putra Malaysia, 43400 Serdang, Selangor, Malaysia; ^5^Biotechnology Research Centre, Malaysian Agriculture Research Development Institute, 43400 Serdang, Selangor, Malaysia; ^6^Department of Bioprocess Biotechnology, Malaysian Agriculture Research Development Institute, 43400 Serdang, Selangor, Malaysia

## Abstract

Legumes have previously been reported with hypolipidemic effect caused by the presence of flavonoid. This study was carried out to evaluate the antioxidant and hypolipidemic effects of fermented mung bean on hypercholesterolemic mice. Blood from all mice was collected and subjected to serum lipid and liver profiles biochemical analysis and quantitative RT-PCR for atherosclerosis related gene expressions. Besides, livers were collected for antioxidant assays and histopathology evaluation. Fermented mung bean was found to reduce the level of serum lipid and liver enzyme profiles of hypercholesterolemic mice. Furthermore, liver antioxidant and nitric oxide levels were also significantly restored by fermented mung bean in a dosage dependent manner. The gene expression study indicated that *Apoe* and *Bcl2a1a* were upregulated while *Npy* and *Vwf* expressions were downregulated after the treatment. The effects of fermented mung bean were greater than nonfermented mung bean. These results indicated that fermented mung bean possessed antioxidants that lead to its hypolipidemic effect on hypercholesterolemic mice.

## 1. Introduction

Hypercholesterolemia is generally caused by high-cholesterol diet and the lack of physical exercise. High income or developed countries such as USA and Western Europe have recorded higher incidence of hypercholesterolemia in their population due to the prevalence of raised cholesterol levels [1, 2]. This has been correlated to higher risk of cardiovascular disease and stroke. Besides, high cholesterol diet also contributed to steatohepatitis, which is often characterized by inflammation and mitochondria glutathione depletion [3]. Nowadays, the trend to reduce hypercholesterolemia through consuming functional foods has gained more acceptances.

Legumes such as soybean and mung bean have been identified as potential functional foods for the prevention of chronic diseases including cancer and cardiovascular diseases. For example, soy protein and isoflavones have been approved by the US Food and Drug Administration to be used as health foods for control of triglyceride (TG) and total cholesterol levels [4]. Mung bean (*Vigna radiata*) is a legume that contains large amounts of protein, essential amino acids, and metabolites such as *γ*-amino butyric acid (GABA) [5]. In Chinese medicine, mung bean has been widely used to prepare mung bean soup for cooling and detoxification of the body [6]. Moreover, mung bean was found to be a potent antioxidant and hepatoprotective agent [5]. Our earlier research has reported the potential of fermentation in improving these effects shown by the mung bean [5]. Besides, fermented mung bean was also found to be a good source of GABA [5], while GABA enriched foods including tempeh (fermented soy) [7] and brown rice bran [8] have been recognized as good sources of functional foods to reduce high lipid profiles. To date, the hypolipidemic and hepatoprotective effects of fermented mung bean on hypercholesterolemic mice are still unknown. In this study, we evaluated its hypolipidemic and hepatoprotective effects through* in vivo* serum biochemical profiles, liver antioxidant level, and blood atherosclerosis related gene expressions of fermented mung bean treated hypercholesterolemic mice.

## 2. Materials and Methods

### 2.1. Materials

Cholesterol, phosphate buffer saline (PBS), Folin-Ciocalteu reagent, aluminium chloride, sodium nitrate, hypoxanthine, xanthine oxidase, and superoxide dismutase were purchased from Sigma (USA). Positive control red yeast rice (HypoCol) containing 2% monacolin k/100 mg of capsule was purchased from AsiaPharm Biotech (Singapore). Total cholesterol, triglyceride, low density lipoprotein, and high density lipoprotein assay kits were purchased from Biovision (USA), Griess reagent was purchased from Invitrogen (USA), and RNeasy mini kit was purchased from Qiagen (USA), while cDNA first-strand synthesis kit and atherosclerosis RT^2^ PCR array were purchased from SABiosciences (USA).

### 2.2. Preparation of Fermented Mung Bean

Fermented mung bean was prepared using* Monascus purpureus* strains (Malaysian Agricultural Research and Development Institute, Malaysia) based on our previous publication. Briefly, mung bean (*Vigna radiata*) was dehulled and soaked in water for 18 h at room temperature. Then, the mung bean was washed, steamed (40 minutes), chilled to room temperature, and mixed with Mardi* Rhizopus* sp. strain of 5351 inoculums for 48 h at 30°C. After the inoculation, the fermented mung bean was dried and both fermented and nonfermented mung beans were extracted with deionized water (1 : 20 ratio) at 25°C for 1 h. The water extract was then subjected to freeze drying (at ~−50°C) to yield 25% w/w of extract powder. The GABA concentration in 100 g of dried fermented mung bean extract powder was 0.122 g while the nonfermented mung bean did not contain any GABA [5]. The total phenolic content of fermented and nonfermented mung bean was 38.39 and 11.62 mg gallic acid equivalent/g extract dry weight with protocatechuic acid as the main detected soluble phenolic acid in fermented mung bean (201.32 *μ*g/g extract) and p-coumaric acid in nonfermented mung bean (8.97 *μ*g/g extract) (results not shown). Both freeze-dried fermented and nonfermented mung bean powders were dissolved in normal saline at a concentration of 1 g/mL for feeding use in the following* in vivo* experiment.

### 2.3. Animals and* In Vivo* Experiment

Eight-week-old male Balb/c mice (*n* = 48) were purchased from the Institute of Bioscience, Universiti Putra Malaysia, and housed under 22°C (12 hours light/dark) with standard pellet and drinking water* ad libitum* according to the guidelines from National Institute of Health for Care and Use of Laboratory Animals. This work was approved by the Animal Care and Use Committee, Faculty of Veterinary Medicine (UPM/FPV/PS/3.2.1.551/AUP-R168). Mice were randomly assigned into six groups (*n* = 8) with mice from groups 2 to 6 being fed p.o. with cholesterol (1 g/kg body weight) while group 1 was fed with PBS for 10 weeks. Treatments were started on week 8 where mice from group 3 were fed orally with 60 mg/kg body weight of Hypocol; groups 4 and 5 were fed orally with 200 mg/kg and 1000 mg/kg body weight of fermented mung bean; group 6 was fed orally with 1000 mg/kg body weight of nonfermented mung bean until week 10. Group 2 (negative control) was fed with PBS until week 10. At the end of the experiments, the mice were anesthetized with ether and sacrificed by cervical dislocation. Blood was collected for gene expressions study and serum was obtained for biochemical analysis. Livers were harvested and either fixed for histopathological evaluation or homogenated in PBS for antioxidant quantifications. Serum total cholesterol, TG, LDL, HDL, AST, ALT, and ALP levels were quantified according to the standard protocols from the kits (Biovision, USA) and were measured using a Hitachi 902 Automatic Biochemical Analyzer (Roche, German). On the other hand, SOD, MDA, FRAP, and NO levels from liver homogenates were quantified according to [5]. Furthermore, livers were harvested, fixed, stained with haematoxylin and eosin (H&E), and viewed under bright-field microscopy (Nikon, Japan) according to [5].

Total RNA was isolated from whole blood of groups 2, 3, 5, and 6 using the RNeasy mini kit (Qiagen, USA). cDNA was synthesized using first-strand kit (SABiosciences, USA) and expression of atherosclerosis related genes was profiled using mouse atherosclerosis RT^2^ Profiler PCR array (SABiosciences, USA) according to the manufacturer's protocol using an iCycler iQ real-time PCR system (Bio-Rad, USA). The results were analyzed using the comparative Cq method with normalization of the results with five housekeeping genes included in this PCR array kit. The relative expression (fold change) was calculated by dividing the normalized data of the genes from samples of group 3 or group 5 with the normalized data of the genes from samples of the untreated group 2. Only fold expression changes larger than ±2 were recorded as significant.

### 2.4. Statistical Analysis

The data were presented as mean ± S.D. Significant levels (*P* < 0.05) of treatment and control were analysed using one-way ANOVA followed by Duncan test.

## 3. Results

After 8 weeks of administration with cholesterol (1 g/kg body weight), mice from groups 2–6 were observed with similar body weights and cholesterol levels (body weight = group 2: 36.41 ± 2.75 g; group 3: 35.84 ± 1.77 g; group 4: 36.11 ± 2.34 g; group 5: 35.16 ± 2.54 g; group 6: 35.66 ± 3.24 g; serum cholesterol level = group 2: 201.13 ± 11.87 mg/dL; group 3: 208.14 ± 10.27 mg/dL; group 4: 206.23 ± 12.99 mg/dL; group 5: 205.86 ± 15.74 mg/dL; group 6: 209.61 ± 17.21 mg/dL) while control mice free from cholesterol were observed with body weights of 29.39 ± 1.88 g and serum cholesterol level of 99.54 ± 10.31 mg/dL. At week 10 of cholesterol feeding, untreated mice (group 2) were recorded with higher serum lipid profile (total cholesterol, TG, and LDL) and liver enzyme (AST, ALT, and ALP) levels than normal control mice (group 1). Overall, after 2 weeks of treatment, positive control (group 3) treated groups showed better lipid profile regulation than fermented mung bean. Nonetheless, fermented mung bean was able to regulate the lipid profile of hypercholesterolemic mice in a dosage dependent manner where 1000 mg/kg of fermented mung bean showed higher reduction percentage of cholesterol, TG, and LDL level than 200 mg/kg of fermented mung bean. Unlike the trend observed in the serum lipid profile, the mice liver profiles showed a reversed trend where fermented mung bean treated groups improved better as compared to both the untreated and the Hypocol treated groups. Nonfermented mung bean at 1000 mg/kg showed similar effect as the low concentration of fermented mung bean in lowering of serum lipid and liver profiles.

Hypercholesterolemic mice were recorded with lower antioxidant levels but elevated lipid peroxidation and nitric oxide levels as compared to normal mice. Hypocol and fermented mung bean were able to reduce liver lipid peroxidation levels and improve the antioxidant levels. On the other hand, Hypocol was found to increase the antioxidant levels better than fermented mung bean. However, fermented mung bean was able to reduce the NO level more significantly than the Hypocol group in a dosage dependent manner. Low concentration of fermented mung bean showed similar antioxidant effect to the 1000 mg/kg of nonfermented mung bean.

**Figure 1:**
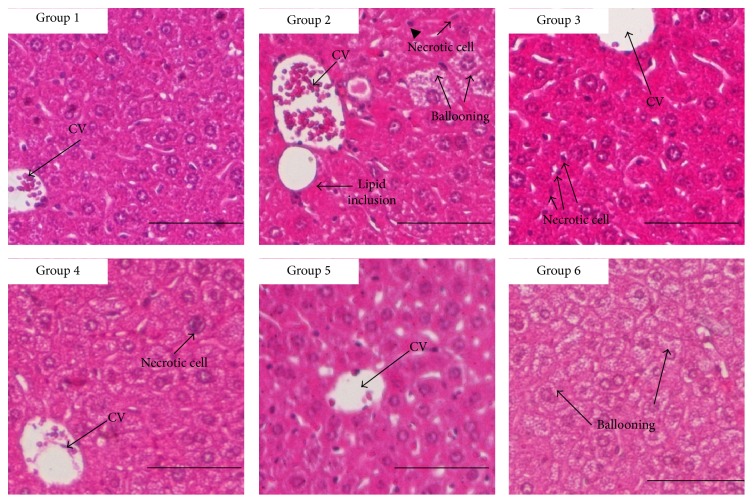
Liver histopathology of group 1 (normal control), 2 (untreated hypercholesterolemic), 3 (hypocol 60 mg/kg body weight), 4 (fermented mung bean 200 mg/kg body weight), and 5 (fermented mung bean 1000 mg/kg body weight) (100x). CV: centrilobular vein.

Histopathology examinations showed that lipid inclusion and ballooning in liver was observed in untreated hypercholesterolemic mice (Figure 1). Lipid inclusion and ballooning were not observed in Hypocol treated, 1000 mg/kg fermented mung bean treated, and healthy normal groups (Figure 1). However, small loci of necrotic cells were still observed in Hypocol and 200 mg/kg body weight of fermented mung bean groups. Furthermore, the loss of cellular boundary was observed in the Hypocol treated group. Comparatively, treatment with 1000 mg/kg body weight of fermented mung bean gave the best recovery.

**Figure 2:**
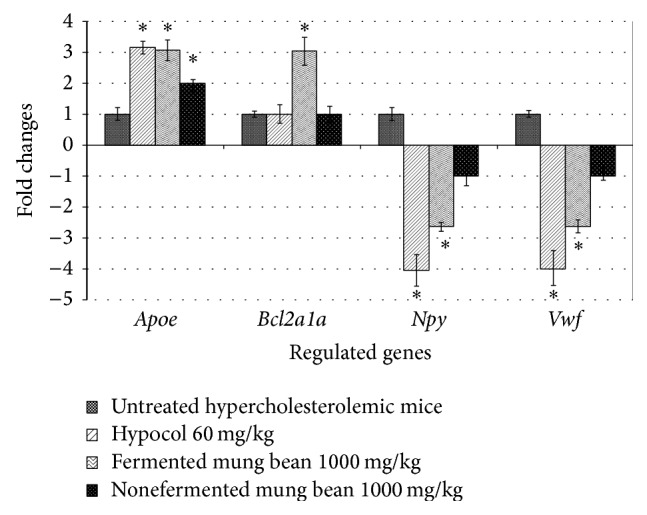
Fold changes of hypocol (60 mg/kg body weight) (*n* = 4) or fermented mung bean (1000 mg/kg body weight) (*n* = 4) positively regulated genes in blood compared to untreated hypercholesterolemic mice (*n* = 4) quantified by real time PCR. The data represent mean and S.D. of 3 independent experiments. Only fold expression changes greater than ±2 were recorded as significant. ^*^Significant difference (*P* < 0.05) among treated or normal group comparing with untreated hypercholesterolemic group was determined using ANOVA followed by Duncan's multiple range test.

To evaluate gene regulation after treatment with Hypocol and fermented mung bean, the expression of atherosclerosis related genes of blood from groups 2, 3, and 5 was tested using mouse atherosclerosis RT^2^ Profiler PCR array (Figure 2). Only 4 genes (*Apoe*,* Bcl2a1a*,* Npy*, and* Vwf*) were positively regulated (>2 fold) in Hypocol and fermented mung bean treatment groups as compared to the untreated hypercholesterolemic mice. Hypocol downregulated high fold changes of* Npy* and* Vwf* genes but similarly upregulated the* Apoe* gene when compared to fermented mung bean. On the other hand, only fermented mung bean treatment groups were able to upregulate* Bcl2a1a* expression in blood.

## 4. Discussion

Increased incidences of cardiovascular diseases globally have been correlated with the burgeoning population with hypercholesterolemia [1]. The consumption of natural foods such as oat-based and fermented products including red yeast rice has been proposed as an alternative method to reduce high cholesterol levels in subjects [9]. However, most of the currently available supplements such as red yeast rice products are not properly standardized and may contain contaminants such as citrinin [10]. Thus, there is a need to identify potential new lipid lowering agents without side effect. Fermented mung bean has been previously reported as antioxidant, hepatoprotective [5], and antihyperglycemic agent [11]. In this study, we have evaluated the hypolipidemic and antioxidant effects of fermented mung bean on hypercholesterolemic mice. Feeding mice with cholesterol has resulted in increased concentrations of serum cholesterol and TG of the untreated mice (Table 1). Previously, we have reported the potential of fermented mung bean to reduce lipid profiles in alloxan-induced diabetic mice [11]. Similar results were obtained in this study where Hypocol and fermented mung bean were found to reduce the level of serum cholesterol, TG, and LDL better than nonfermented mung bean.

Hepatic steatosis is a type of nonalcohol induced fatty liver that is related to hyperlipidemia and obesity [12]. Our results have shown that feeding mice with high cholesterol was associated with ballooning and lipid inclusion in liver histopathology (Figure 2) and drastic increases in AST, ALT, and ALP levels (Table 1). Red yeast rice is not recommended to be consumed by patients with liver problems because it was found to associate with symptomatic hepatitis [13]. In this study, we found that red yeast rice treatment did not improve the liver enzyme profiles and caused abnormal liver histopathology. On the other hand, fermented mung bean which was previously reported as a potential hepatoprotective agent [5] was able to improve the liver enzyme levels and liver histopathology in a dosage dependent manner. Other than hepatic steatosis, high cholesterol was also associated with reduced liver antioxidant levels [14] and increased inflammation in liver, which might subsequently lead to induced hepatocellular death [3]. SOD is the antioxidant enzyme present in liver while FRAP measures the total antioxidant capacity of liver homogenate. MDA is the product of lipid peroxidation while NO is the inflammation mediator found in the liver [5]. In this study, the SOD, FRAP, MDA, and NO levels in liver homogenates were measured. The untreated hypercholesterolemic mice were observed with reducing antioxidant levels and accumulation of inflammatory mediator level (Table 2) in liver homogenates. Red yeast rice was able to increase the antioxidant levels and reduce the lipid peroxides in liver. However, this treatment did not alter the NO level in the liver. The results of liver enzyme profile, liver histology, and liver NO level indicated that inflammation still occurred in the liver of red yeast rice treated mice. Conversely, GABA and fermented mung bean were able to enhance liver's antioxidant activities and reduce lipid peroxidation and inflammatory mediator levels concurrently. This effect in fermented mung bean treated group may be caused by the high phenolic content and the volatile antioxidant present in fermented mung bean [5]. GABA is a nonprotein amino acid that works as a neurotransmitter inhibitor [5]. Previous research has reported GABA as the major component that contributed to the hypocholesterolemic effect of germinated brown rice [15]. On top of that, GABA was also reported to have hepatoprotective effect towards ethanol induced damage [16]. Thus, the presence of GABA in the fermented mung bean [5] may contribute to both the hypocholesterolemic and hepatoprotective effects. Thus GABA present in fermented mung bean may also contribute to reducing the lipid and protecting the liver damage in fermented mung bean treated hypercholesterolemia mice.

The expressions of atherosclerosis related genes in blood were evaluated using real time PCR array (SABiosciences, USA). Only expressions with fold changes greater than 2 (comparing groups 3, 5, and 6 with the untreated group 2) are presented in Figure 1. The hypocholesterolemic effect of fermented mung bean was found to be associated with upregulation of the genes* ApoE* and* Bcl2a1a* but downregulation of* Npy* and* Vwf* in the blood of fermented mung bean treated mice. Apolipoprotein-E (*ApoE*) is a carrier for HDL that removes cholesterol from cell to liver.* ApoE* deficiency was associated with increase of blood cholesterol level and risk of atherosclerosis [17]. The results from the gene expression studies also indicated that red yeast rice and fermented mung bean treatments upregulated* ApoE* to transport cholesterol to liver, which resulted in the reduction of total cholesterol in the blood serum.* Bcl2a1a* is a member of* Bcl2* family that functions as an antiapoptotic protein [18]. The expression of* Bcl2a1a* was only significant in fermented mung bean treated mice. This phenomenon indicated that fermented mung bean may contribute to the protective effect of hepatocyte via upregulation of this antiapoptotic gene that was not observed in red yeast rice treated mice. Neuropeptide Y (*Npy*) is a 36-amino-acid peptide that is highly produced in obese mice [19]. Stimulation of* Npy* increases the appetite and reduces energy expenditure which ends up with promoting more energy storage [20]. Suppression of* Npy* by red yeast rice and fermented mung bean may reduce food intake and increase energy expenditure which indirectly improve the lipid profile of the hypercholesterolemic mice. Von Willebrand factor (*Vwf*) is a procoagulant glycoprotein that promotes platelet adhesion during vascular injury. Feeding mice with cholesterol was found to elevate the plasma level of* Vwf* which is now being used as one of the indicators of endothelial damage in vascular disease [21]. Suppression of* Vwf* expression was observed in red yeast rice and fermented mung bean treated hypercholesterolemic mice. These indicate that both treatments successfully reduced cholesterol levels in mice and subsequently reduced the level of endothelial damage and the risk of vascular disease.

In conclusion, fermented mung bean showed comparable hypolipidemic effect as red yeast rice through upregulation of* ApoE* and downregulation of* Npy* expressions. Moreover, its antioxidant activities were also able to reduce the high cholesterol associated hepatic steatosis and inflammation through upregulation of the* Bcl2a1a* antiapoptotic gene. However, further studies are needed on the genes that regulate anti-inflammation (e.g.,* NFkB*), antioxidant (e.g.,* Nrf2*), antiapoptotic (*Bcl2a1a*), and antiatherosclerosis (*Npy* and* ApoE*) mechanisms using Western blot in short- and long-term fermented mung bean treated hypercholesterolemic mice to validate its hypolipidemic effects and its ability to reduce the high cholesterol associated hepatic steatosis and inflammation.

## Figures and Tables

**Table 1:** Blood serum lipid and liver profiles of hypercholesterolemic mice after 2 weeks of treatments.

Treatment	Cholesterol (mg/dL)	Triglyceride (mg/dL)	LDL (mg/dL)	HDL (mg/dL)	ALT (U/L)	ALP (U/L)	AST (U/L)
Group 1 (*n* = 8)	103.71 ± 9.13^*^	101.52 ± 14.38^*^	39.00 ± 2.39^*^	57.86 ± 4.25^*^	62.50 ± 2.54^*^	90.13 ± 3.23^*^	120.42 ± 11.05^*^
Group 2 (*n* = 8)	216.73 ± 12.99	201.14 ± 12.52	86.18 ± 2.93	58.52 ± 3.19	277.20 ± 8.71	127.83 ± 5.21	320.13 ± 13.82
Group 3 (*n* = 8)	152.72 ± 7.36^*^	155.41 ± 9.84^*^	57.16 ± 1.38^*^	74.51 ± 3.42^*^	263.58 ± 4.18	137.00 ± 2.74	424.42 ± 14.07^*^
Group 4 (*n* = 8)	168.22 ± 5.41^*^	142.10 ± 11.85^*^	66.14 ± 6.48^*^	69.03 ± 5.65^*^	190.05 ± 2.81^*^	109.00 ± 2.77^*^	303.16 ± 9.11
Group 5 (*n* = 8)	163.41 ± 7.02^*^	133.20 ± 9.27^*^	59.46 ± 4.86^*^	71.51 ± 7.72^*^	162.50 ± 4.00^*^	102.75 ± 1.50^*^	186.72 ± 16.29^*^
Group 6 (*n* = 8)	181.75 ± 5.21^*^	172.13 ± 8.15^*^	71.11 ± 3.74^*^	66.28 ± 4.11^*^	181.58 ± 5.32^*^	110.36 ± 2.89^*^	294.61 ± 5.62^*^

^*^Significant difference (*P* < 0.05) among treated or normal group comparing with untreated hypercholesterolemic group was determined using ANOVA followed by Duncan's multiple range test.

**Table 2:** Liver homogenate antioxidant and nitric oxide levels of hypercholesterolemic mice after 2 weeks of treatments.

Treatment	MDA (nM MDA/mg sample)	FRAP (*μ*M Fe(II)/mg of protein)	SOD (unit SOD/mg sample)	NO (*μ*M/mg of protein)
Group 1 (n = 8)	0.72 ± 0.15^*^	2.03 ± 0.51^*^	0.90 ± 0.12^*^	7.52 ± 1.13^*^
Group 2 (n = 8)	2.21 ± 0.13	3.80 ± 0.31	0.60 ± 0.01	21.37 ± 0.53
Group 3 (n = 8)	0.86 ± 0.01^*^	2.56 ± 0.44^*^	0.90 ± 0.15^*^	18.72 ± 1.62^*^
Group 4 (n = 8)	1.19 ± 0.55^*^	1.36 ± 0.15^*^	0.66 ± 0.13^*^	17.45 ± 0.89^*^
Group 5 (n = 8)	1.06 ± 0.34^*^	2.05 ± 0.56^*^	0.78 ± 0.04^*^	13.11 ± 1.74^*^
Group 6 (n = 8)	1.23 ± 0.27^*^	1.42 ± 0.57^*^	0.64 ± 0.36^*^	18.21 ± 2.10^*^

^*^Significant difference (*P* < 0.05) among treated or normal group comparing with untreated hypercholesterolemic group was determined using ANOVA followed by Duncan's multiple range test.
